# Gastrin-releasing peptide, neuromedin B, and symptom severity in adult attention-deficit hyperactivity disorder

**DOI:** 10.1590/1806-9282.20260659

**Published:** 2026-10-12

**Authors:** Elif Özcan Tozoğlu, Alev Lazoğlu Özkaya

**Affiliations:** 1 University of Health Sciences, Erzurum Faculty of Medicine, Department of Psychiatry – Erzurum, Türkiye.; 2 Erzurum City Hospital, Department of Biochemistry – Erzurum, Turkey.

**Keywords:** Attention deficit disorder with hyperactivity, Biomarkers, Gastrin-releasing peptide, Neuromedin b, Neuropeptides

## Abstract

**OBJECTIVE:**

Attention-deficit hyperactivity disorder is a neurodevelopmental disorder associated with metabolic and neuropeptidergic processes. Gastrin-releasing peptide and neuromedin B, which are bombesin-like peptides, have been studied only to a limited extent in adult attention-deficit hyperactivity disorder, despite their role in energy homeostasis and central nervous system function. The aim of this study was to evaluate gastrin-releasing peptide and neuromedin B levels in untreated adult attention-deficit hyperactivity disorder patients and to examine their relationship with clinical symptom severity.

**METHODS:**

This cross-sectional study included 55 untreated adult attention-deficit hyperactivity disorder patients and 32 healthy controls. Attention-deficit hyperactivity disorder diagnoses were confirmed using SCID-5/CV and DIVA 2.0, and symptom severity was assessed using the Adult Attention-Deficit Hyperactivity Disorder Self-Report Scale. Gastrin-releasing peptide and neuromedin B levels in fasting serum were measured using the ELISA method. Group comparisons, Pearson correlations, hierarchical regression, and receiver operating characteristic analyses were performed.

**RESULTS:**

Gastrin-releasing peptide (22.83±24.18 pg/mL) and neuromedin B (1.83±2.86 pg/mL) levels were significantly lower in the attention-deficit hyperactivity disorder group compared to the control group (p=0.004 and p=0.037, respectively). Gastrin-releasing peptide and neuromedin B levels were negatively correlated with Attention-Deficit Hyperactivity Disorder Self-Report Scale total and subscale scores (p<0.001 for all). In regression analyses, gastrin-releasing peptide was an independent predictor of Attention-Deficit Hyperactivity Disorder Self-Report Scale total score (β=-0.642, p=0.024). In receiver operating characteristic analyses, gastrin-releasing peptide (AUC=0.746) and neuromedin B (AUC=0.719) showed significant performance in distinguishing attention-deficit hyperactivity disorder.

**CONCLUSION:**

The findings suggest that gastrin-releasing peptide, in particular, may be associated with clinical symptom severity in adult attention-deficit hyperactivity disorder and is a potential biomarker candidate.

## INTRODUCTION

Attention-deficit hyperactivity disorder (ADHD) is a neurodevelopmental disorder characterized by inattention, hyperactivity, and impulsivity, which may persist into adulthood. Its etiology is thought to involve complex interactions between genetic, environmental, and neurobiological factors^1^.

Recent research has focused on the role of metabolic processes and energy homeostasis in neurodevelopmental disorders, suggesting that appetite-related neuropeptides may contribute to behavioral and neurobiological mechanisms associated with ADHD.

Bombesin-like peptides, including gastrin-releasing peptide (GRP) and neuromedin beta (NMB), are anorexigenic neuropeptides that regulate metabolic and neurobehavioral processes via specific G-protein-coupled receptors^2,3^. Experimental studies suggest that GRP and NMB are involved in metabolic regulation and energy balance^4–6^.

In addition to their metabolic functions, GRP and NMB are involved in central nervous system processes related to stress responses, synaptic plasticity, and behavioral regulations^7,8^. Clinical studies in autism have reported improvements in symptoms such as hyperactivity and social functioning following GRP administration^9,10^. Moreover, experimental findings indicate that GRP can stimulate dopamine and norepinephrine release, both of which are neurotransmitter systems associated with ADHD^11^.

These neuropeptides are also associated with metabolic and inflammatory processes, including glucose metabolism, lipid regulation, and immune responses^12–14^. Therefore, evaluating GRP and NMB levels alongside metabolic parameters may contribute to a more comprehensive understanding of homeostatic mechanisms.

Despite growing interest in neuropeptide-based mechanisms, there are no clinical studies evaluating GRP and NMB levels together in untreated adult ADHD populations. Therefore, the present study aimed to investigate GRP and NMB levels in adults with ADHD and examine their relationship with symptom severity while controlling for potential metabolic and inflammatory confounders.

## METHODS

### Ethics approval and consent to participate

Ethical approval for this study was obtained from the Erzurum Faculty of Medicine Scientific Research Ethics Committee, University of Health Sciences (Decision No: 2024/12-216). The study was conducted in accordance with the Declaration of Helsinki, and written informed consent was obtained from all participants.

### Research sample

The sample consisted of untreated adult ADHD patients who presented to the Psychiatry Outpatient Clinic of Erzurum City Hospital between December 2024 and May 2025, and age- and gender-matched healthy controls without psychiatric disorders.

Sample size was calculated using G*Power 3.1 (α=0.05, power=0.80, effect size d=0.5), indicating a minimum of 64 participants.

### Participant selection and exclusion criteria

The study included adults aged 18–65 diagnosed with ADHD who had not received prior psychiatric treatment, and healthy controls matched for age and gender. ADHD diagnosis was confirmed using SCID-5/CV and DIVA 2.0, and individuals with additional psychiatric disorders were excluded.

To minimize confounding metabolic and inflammatory effects on GRP and NMB levels, the following exclusion criteria were applied: BMI ≥30 kg/m^2^, history of diabetes, hypertension, liver or renal disease, active infection, use of lipid-lowering medications, and severe dyslipidemia (LDL>190 mg/dL or triglycerides>400 mg/dL). Mild-to-moderate lipid elevations below these thresholds were allowed and controlled for as covariates.

### Procedure

This cross-sectional study evaluated clinical characteristics, biochemical parameters, and GRP and NMB levels. Symptom severity was assessed using the Adult ADHD Self-Report Scale (ASRS), and sociodemographic data were collected via a standardized form.

Venous blood samples were collected between 8:00 and 10:00 a.m. after an 8–12-h overnight fast. Participants were instructed to avoid alcohol, caffeine, and intense physical activity prior to sampling. Blood samples from female participants were collected between days 2 and 5 of the menstrual cycle to minimize hormonal variability.

### Data collection tools

Sociodemographic data were collected using a structured form. Psychiatric diagnoses were assessed using SCID-5/CV, and ADHD diagnosis was supported by DIVA 2.0^15^. Symptom severity was evaluated using the ASRS^16,17^.

### Biochemical analysis

Serum samples were obtained following centrifugation and stored at −80°C until analysis. Routine biochemical parameters (ALT, AST, GGT, glucose, lipid profile, HbA1c) were measured using standard automated laboratory methods.

GRP and NMB levels were determined using commercially available ELISA kits according to manufacturer protocols.

### Statistical analysis

Data were analyzed using SPSS 26.0. Continuous variables were expressed as mean±SD, and categorical variables as frequencies and percentages. Group comparisons were performed using independent samples t-tests and chi-square tests.

Pearson correlation analysis was used to assess relationships between biomarkers and symptom severity. Hierarchical multiple regression analyses were conducted to evaluate predictors of ADHD symptoms, with age, gender, smoking, LDL, HbA1c, and C-reactive protein (CRP) entered in the first block and GRP and NMB in the second block.

Receiver operating characteristic (ROC) analysis was used to evaluate the diagnostic performance of GRP and NMB; area under the curve (AUC), optimal cutoff values (Youden index), sensitivity, and specificity were calculated. A p-value<0.05 was considered statistically significant.

## RESULTS

A total of 87 participants were included in the study, comprising 55 ADHD patients and 32 healthy controls. This sample size was found to meet the minimum requirement determined by G*Power. The sociodemographic data of the participants are presented in Table 1. There was no significant difference between the ADHD and control groups in terms of age and gender (p>0.05).

**Table 1:** Sociodemographic and biochemical characteristics of the study groups.

Variable		ADHD (n, mean±SD)	Control (n, mean±SD)	p-value/χ^2^
Age		32.01	32.96	0.718
Gender	Female	25	14	0.877
	Male	30	18	
Education	Primary education	17	0	0.001
	High school	15	8	
	University	23	24	
Marital status	Married	28	22	0.267
	Single	24	9	
	Divorced/widowed	3	1	
Occupation/employment status	Worker-employee	26	26	0.008
	Student	4	2	
	Housewife	17	1	
	Not working	8	3	
Family history of mental illness	There	4	1	0.423
	None	51	31	
Smoking status	Yes	37	13	0.015
	None	18	19	
GRP		22.83±24.18	41.99±30.39	0.004
NMB		1.83±2.86	3.51±3.87	0.037
ALT		26.07±11.28	25.53±11.75	0.834
AST		17.00±7.03	14.50±3.98	0.037
GGT		21.96±11.29	22.90±15.78	0.768
TG		115.60±53.10	102.97±44.17	0.237
HDL		42.43±10.35	43.22±10.96	0.743
LDL		112.69±37.32	120.63±33.70	0.312
Cholesterol		161.95±29.87	172.50±32.05	0.134
FBG		80.95±12.15	83.62±10.29	0.277
HbA1c		4.91±0.57	5.23±0.42	0.004
CRP		2.96±2.09	2.83±1.50	0.729

GRP: gastrin-releasing peptide; NMB: neuromedin B; ALT: alanine aminotransferase; AST: aspartate aminotransferase; FBG: fasting blood glucose; HbA1c: hemoglobin A1c; HDL: high-density lipoprotein; LDL: low-density lipoprotein; TG: triglyceride; ADHD: attention-deficit hyperactivity disorder; SD: standard deviation; GGT: gamma-glutamyl transferase; CRP: C-reactive protein; ROC: receiver operating characteristic.

When the biochemical measurements of the groups were evaluated, GRP and NMB levels were found to be significantly lower in the ADHD group compared to the control group (p=0.004 and p=0.037, respectively; Table 1). Pearson correlation analysis revealed a strong positive correlation between GRP and NMB levels (r=0.914, p<0.001). Both biomarkers were significantly and negatively correlated with ASRS total and subscale scores (all p<0.001), indicating that lower biomarker levels were associated with greater symptom severity.

In hierarchical multiple regression analyses conducted to evaluate the predictors of ADHD symptoms (Table 2), the model created for the ASRS total score, with age, gender, smoking, LDL, HbA1c, and CRP in the first block, explained 35.9% of the variance. Adding GRP and NMB in the second block significantly improved the model and increased the total variance explained to 52.5%. In this model, only GRP was found to be a significant predictor (β=-0.642, p=0.024), while the independent effect of NMB was not significant.

**Table 2:** Hierarchical multiple regression results.

Outcome	Model	R	Adj. R^2^	ΔR^2^	ΔF (p)	β (GRP)	p (GRP)	β (NMB)	p (NMB)
ASRS total	Block 1	0.359	0.284	–	–	–	–	–	–
	Block 2	0.525	0.441	0.165	8,008 (0.001)	-0.642	0.024	0.071	0.777
ASRS attention	Block 1	0.342	0.265	–	–	–	–	–	–
	Block 2	0.537	0.462	0.195	9,709 (<0.001)	-0.931	0.001	-0.210	0.147
ASRS hyperactivity	Block 1	0.296	0.214	–	–	–	–	–	–
	Block 2	0.413	0.330	0.116	4,551 (0.016)	-0.276	0.426	-0.207	0.404

Notes: Block 1 covariates: Age, Sex, Smoking status, LDL, HbA1c, CRP. Block 2 predictors: GRP and NMB. ΔR^2^: change in explained variance. β: standardized coefficient. Adj. R^2^: adjusted R^2^. ΔR^2^: change in explained variance after adding Block 2 variables. β: standardized regression coefficient. Adj. R^2^: adjusted R-squared. p<0.05 was considered statistically significant. NMB: neuromedin B; ASRS: Attention-Deficit Hyperactivity Disorder Self-Report Scale.

The analysis for the attention-deficit subscale showed that the first block explained 34.2% of the variance. Adding GRP and NMB in the second block significantly increased the variance explained, and the total variance explained reached 53.7%. In this subscale, GRP was a strong predictor (β=-0.931, p=0.001), while the effect of NMB was not significant.

In the model created for hyperactivity/impulsivity, the first block explained 29.6% of the variance in symptoms. Adding GRP and NMB in the second block led to a significant improvement in the model, and the total variance increased to 41.3%. However, in this subscale, the independent effects of both GRP and NMB were not statistically significant.

The performance of GRP and NMB levels in distinguishing ADHD from the control group was evaluated using ROC analysis (Figure 1). The optimal cutoff value for GRP was 17.6 pg/mL, showing 84% sensitivity and 65% specificity. The optimal cutoff value for NMB was determined to be 0.905 pg/mL, providing 78% sensitivity and 64% specificity. Both biomarkers were found to have significant diagnostic power in distinguishing ADHD diagnoses.

**Figure 1:**
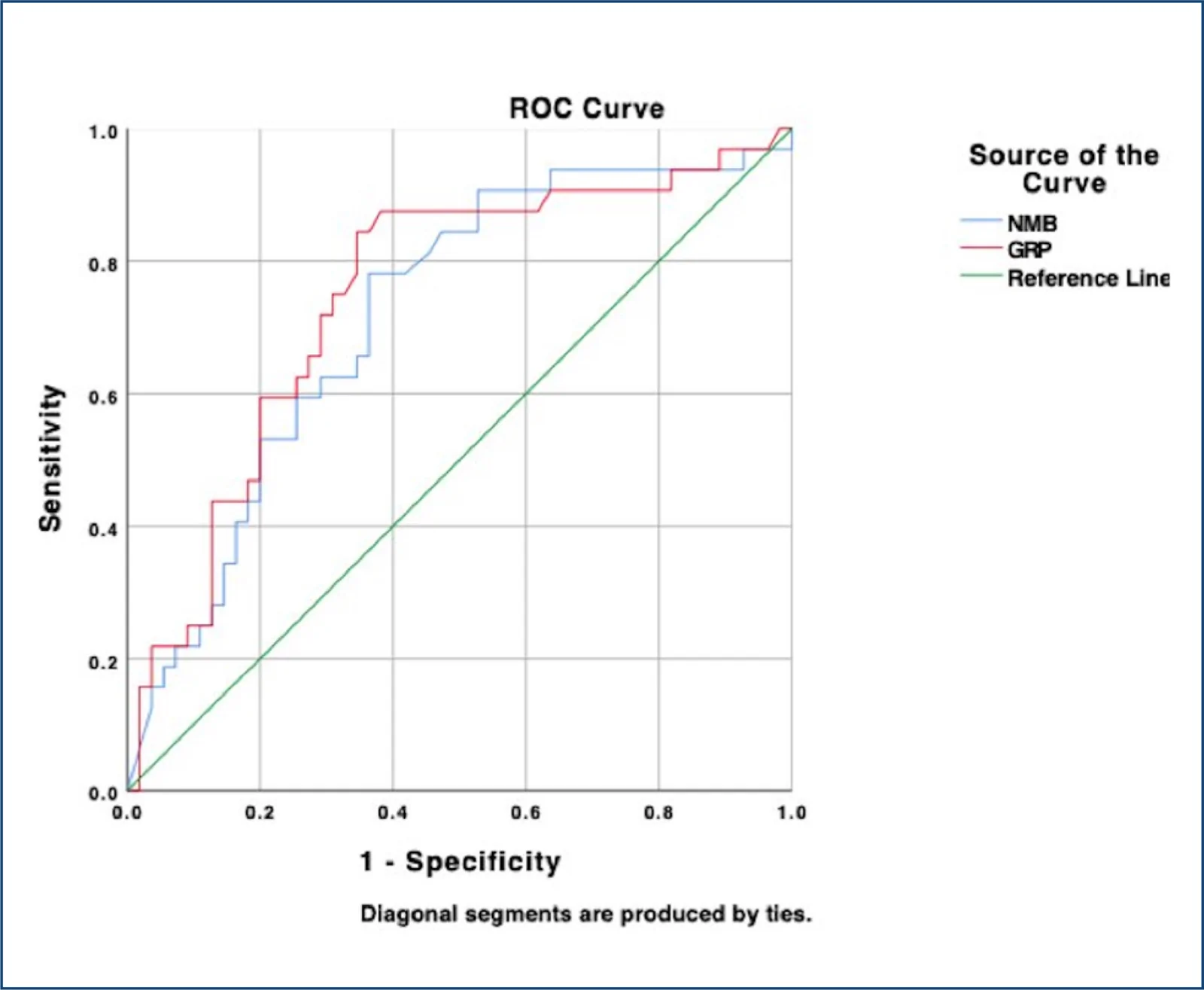
ROC analysis to distinguish attention-deficit hyperactivity disorder patients from healthy controls using gastrin-releasing peptide and neuromedin B levels. GRP: 95%CI 0.636–0.856, area: 0.746, standard error: 0.056, p<0.001. The optimal cutoff value determined by the maximum Youden index was 17.6, yielding a sensitivity of 84.4% and a specificity of 65.5%. NMB: 95%CI 0.608–0.830, area: 0.719, standard error: 0.057, p=0.001. The optimal cutoff value was 0.905, providing a sensitivity of 78.1% and a specificity of 63.6%.

## DISCUSSION

This study found that GRP and NMB levels were significantly lower in untreated adult ADHD patients compared to healthy controls. Both biomarkers were negatively associated with ADHD symptom severity, particularly ASRS total and subscale scores, with symptoms of inattention and hyperactivity/impulsivity increasing as levels decreased. Hierarchical regression analyses showed that GRP was an independent predictor of ADHD symptom severity even after controlling for demographic and metabolic variables. ROC analyses showed that both GRP and NMB demonstrated significant discriminatory performance in distinguishing ADHD from healthy controls.

Taken together, these findings support the hypothesis that bombesin-like peptides may contribute to the biological heterogeneity of adult ADHD beyond traditional catecholaminergic mechanisms.

To our knowledge, this is the first clinical study to jointly evaluate circulating GRP and NMB levels in untreated adult ADHD patients, providing novel insights into the metabolic and neuropeptidergic underpinnings of ADHD.

The rationale for this study was based on the absence of clinical evidence regarding the combined evaluation of circulating GRP and NMB in untreated adults with ADHD, despite growing evidence linking bombesin-like peptides to metabolic regulation and central neurotransmitter systems. By examining these peptides together while controlling for major metabolic and inflammatory confounders, we sought to clarify whether they are associated with ADHD symptom severity and whether they may represent biologically relevant markers of the disorder.

Recent studies have emphasized that ADHD is not limited to catecholaminergic dysfunction, but also involves metabolic processes and energy homeostasis. Metabolomic studies have revealed alterations in amino acid metabolism, neurotransmitter precursors, and lipid pathways, suggesting that peripheral metabolic signals may reflect central nervous system dysfunction^18,19^. In this context, the lower GRP and NMB levels observed in our study, along with their negative associations with symptom severity, are consistent with evidence indicating that peripheral metabolic dysregulation is linked to clinical manifestations of ADHD^18,19^.

Evidence regarding appetite-related hormones further supports this framework. Previous studies have reported alterations in peptides such as AgRP and asprosin, as well as associations between hormones like leptin and ghrelin and ADHD symptoms^20–22^. The reduced GRP and NMB levels observed in our study align with this broader neuropeptidergic network and suggest that bombesin-like peptides may also contribute to the biological basis of ADHD.

The literature indicates that metabolic and neuropeptidergic markers may show significant associations with ADHD symptoms, but these relationships are not always maintained in multivariate models^18,19^. Consistent with this, NMB was associated with symptom severity in correlation analyses but did not emerge as an independent predictor in regression models.

In contrast, GRP remained an independent predictor even after controlling for covariates. Although GRP and NMB belong to the same peptide family, differences in their biological roles may explain this divergence. Metabolomic studies have shown that peripheral metabolites related to dopaminergic and noradrenergic pathways are associated with ADHD symptoms and executive functions^18,19,23^, suggesting that peripheral signals may reflect central neurotransmitter processes. In this context, GRP may have a closer biological relationship with these pathways, which could explain its stronger association with symptom severity.

Although biomarker research in ADHD is increasing, it is widely accepted that single markers are insufficient for diagnosis in such a heterogeneous disorder. Multimodal and multi-marker approaches have been shown to provide better diagnostic performance^23,24^. While GRP and NMB demonstrated significant discriminatory performance in our ROC analyses, their clinical utility as standalone diagnostic markers remains limited. Rather, these findings contribute to understanding the biological mechanisms of ADHD and may inform future biomarker-based approaches.

From a clinical perspective, GRP and NMB are not intended to replace current diagnostic procedures for ADHD. However, these biomarkers may complement future multimodal assessment strategies by providing objective biological information related to symptom severity and underlying neurobiological mechanisms. If confirmed in larger longitudinal studies, GRP in particular may help identify biologically distinct ADHD subgroups or serve as a potential biomarker for treatment monitoring and individualized therapeutic approaches.

This study has several important strengths. First, only treatment-naïve adults with ADHD were included, eliminating the potential influence of psychotropic medications on circulating peptide levels. Second, strict exclusion criteria minimized the effects of metabolic and inflammatory conditions that could affect GRP and NMB concentrations. Third, demographic, metabolic, and inflammatory variables were statistically controlled using hierarchical regression analyses, allowing the independent contribution of these neuropeptides to be evaluated. Finally, to our knowledge, this is the first clinical study to simultaneously investigate circulating GRP and NMB levels in untreated adults with ADHD.

Nevertheless, several limitations should be acknowledged. Although the sample size was sufficient according to the a priori power analysis, larger multicenter studies are needed to improve the generalizability of these findings. The cross-sectional design precludes causal inferences regarding the relationship between GRP, NMB, and ADHD symptom severity. In addition, the single-center design may limit the external validity of the findings, and residual hormonal variability in female participants cannot be completely excluded despite efforts to standardize blood sampling. Furthermore, serum GRP and NMB concentrations may not fully reflect their activity within the central nervous system; therefore, the observed associations should be interpreted cautiously until confirmed by studies integrating peripheral biomarkers with central neurobiological measures.

## CONCLUSION

In conclusion, this study demonstrates that circulating GRP and NMB levels are reduced in untreated adults with ADHD and are associated with greater symptom severity. Among these biomarkers, GRP remained an independent predictor of ADHD symptom severity after adjustment for demographic, metabolic, and inflammatory factors, suggesting a potentially more prominent role in the biological mechanisms underlying the disorder. Although these findings are not sufficient to support the clinical use of GRP or NMB as standalone diagnostic biomarkers, they provide further evidence for the involvement of metabolic and neuropeptidergic pathways in ADHD and support future multimodal biomarker research. Larger longitudinal and mechanistic studies are needed to validate these findings and determine their potential role in diagnosis, prognosis, and treatment monitoring.

## Data Availability

The datasets generated and/or analyzed during the current study are available from the corresponding author upon reasonable request.
